# The University of Oxford Diploma in Ophthalmology

**Published:** 1909-06-12

**Authors:** 


### The University of Oxford Diploma in Ophthalmology.

The University of Oxford has decided to institute a diploma in ophthalmology, and has recently announced the conditions under which this diploma will be granted. Certain of these conditions are, in our judgment, open to criticism. Antecedent to them, however, lies the question whether the diploma itself is a development likely to promote or to hinder the interests of the medical profession and of the general public. For our own part, and in answer to this question, we have no hesitation in saying that the decision of the University is much to be deplored. The advantages which may be expected to follow it are of a most restricted order, while its evil effects may be both numerous and widely spread. Into the secluded atmosphere in which academic decisions are believed to be framed the voice of the busy and practical world may hardly hope to penetrate. Nevertheless we shall state the reasons which have led us, after full consideration, to pass an adverse decision on the University policy.

Our first objection to this policy is that, without any corresponding advantage to professional efficiency, it adds yet another paper qualification to a list of ornamental distinctions already far too numerous. No one can affect to believe that the institution of the new diploma and the modest curriculum which it demands will do much, if anything, to raise the general level of the study of ophthalmology in this country. The standard attained by those who restrict their practice to ophthalmic surgery is already a high one, and what is wanted is a more exact knowledge of the subject by the whole body of the profession. The ordinary curriculum now makes some demands in this direction, and these demands might well be increased. But a special diploma, so far from helping to this end, is a direct suggestion that ophthalmology is without the scope of general medicine, and that the knowledge and practice of it are legitimately restricted to a limited number of practitioners. This we regard as an unfortunate and disastrous suggestion, and one which in itself is sufficient to justify opposition to the scheme.

Further, if one University is to confer a diploma in ophthalmology, and this on terms specially favourable to its own graduates, the other Universities will be almost compelled in self-defence to take similar action. Nor is it likely that the movement wlll be restricted to ophthalmology. If this justifies a special diploma a similar claim may be made for neurology, gynæcology, dermatology, otology, laryngology, rhinology, and possibly for other limited departments of medical and surgical practice. The public mind is already sufficiently confused by the existing crowd of medical titles, but if the Oxford lead is to be followed confusion will soon become even worse confounded. Again, it is almost inevitable that with the growth and multiplication of such diplomas there will arise a demand for the restriction of certain departments of practice to the holders of these distinctions. And this without question would not be to the advantage either of those engaged in general practice or of the patients placed in their charge.

Finally, and apart from the general policy of special diplomas, it may be urged that whatever may be true in other cases, ophthalmology is not a subject suitable for recognition in this fashion. In some of its aspects it is purely surgical, and ophthalmic surgery is mainly the application of general surgical principles in a special and limited field. But excepting those local conditions capable of treatment by surgical or mechanical measures, pathological disturbances in the visual apparatus have no relation to ophthalmic surgery. They depend on intra-cranial or general constitutional disease, and the investigation and treatment of them demand the methods and experience not of the surgeon, but of the physician. Ophthalmic surgery has no more to say to them than has orthopædic surgery to say to an attack of acute gout affecting a metatarso-phalangeal joint. Such disturbances belong to the general body of medicine, and they claim not the local measures of surgery, but the wider outlook and general treatment of medical practice. Yet if the Oxford precedent is to be followed only those who study ophthalmic surgery will have a claim to academic recognition as diplomates in ophthalmology. This, in our judgment, is a thoroughly unsound position and is opposed both to scientific and to practical progress. The fact of the matter is that ophthalmology is not a homogeneous or special department either of medicine on the one hand or of surgery on the other, and, therefore, it is quite unsuitable for the recognition of a special diploma, even allowing that the policy of the special diploma is in any circumstances a desirable one.

